# The Clinical Value of Patient Home Videos in Movement Disorders

**DOI:** 10.5334/tohm.651

**Published:** 2021-09-21

**Authors:** Andrew Billnitzer, Joseph Jankovic

**Affiliations:** 1 Parkinson’s Disease Center and Movement Disorders Clinic, Baylor College of Medicine, Department of Neurology, Houston, Texas

**Keywords:** Movement disorders, home videos, phenomenology, mobile technology

## Abstract

**Background:**

Numerous studies have shown the value of patient home video recordings within the field of epilepsy. Despite the growing influence of mobile technology and telemedicine, there is a paucity of studies examining the role of home videos in the diagnosis of movement disorders.

**Objective:**

To characterize the clinical value of patient home videos in a movement disorders practice.

**Methods:**

We performed a retrospective review from our video database over the past 10 years and identified 20 encounters where an in-person, clinic evaluation and studio video were supplemented by a home video. We reviewed these encounters to determine if the home video added additional value to the clinic video. The home videos were screened by 3 movement disorders attendings and 3 movement disorders fellows to assess for quality and to determine whether or not the patient phenomenology could accurately be identified.

**Results:**

Of the 20 videos identified, 10 (50%) were determined to be of additional clinical value. In 62.4% of evaluations movement disorders attendings and fellows were able to identify phenomenology from the home videos consistent with the final diagnosis. Videos rated as “poor” quality had significantly lower odds of leading to a correct phenomenology (odd ratio: 0.07, 95% confidence interval [0.01–0.72]) than those rated as “excellent” quality.

**Conclusions:**

Patients should be encouraged to produce good quality home videos, particularly in paroxysmal or fluctuating movement disorders, as they may add value to the eventual diagnosis and management.

## Introduction

Video recordings have a well-established utility in the medical field as a teaching, diagnostic, and research tool. In a survey of 70 children’s hospitals across Canada, the United Kingdom, and the United states, 94% of hospitals reported using video recording of patients in some capacity [1]. Within neurology, in the field of epilepsy, the benefits of home video recordings have been well established. In a recent study, patient home videos, obtained on a smartphone, increased the chance of correctly diagnosing an epileptic event when compared to chance of obtaining a diagnosis from history and physical examination alone (95.2% vs 78.6%) [2]. In another study in a developing nation, home videos alone, when evaluated by a specialist, were found to have a 97.2% concordance rate with a diagnosis of epileptic seizure or psychogenic non-epileptic seizure when compared to a video EEG gold standard of the same patients [3]. Additionally, home videos have been shown to be a valuable adjunct tool in identifying paroxysmal events in infants, increasing the accuracy of diagnosis of both epileptic events and non-epileptic events by 3.9% and 11.5%, respectively [4].

With the advances in mobile technology there has been an increased implementation of wearables, telemedicine, and other device or computer-based methods in movement disorders practices and research [5,6,7]. In our own movement disorders practice we often rely on patient home videos to better characterize the phenomenology of the movement disorder and to document paroxysmal, intermittent, fluctuating or task-specific features not seen or fully appreciated during clinic encounters. The primary aim of this study was to explore the role of home video recordings in the diagnosis and management of movement disorders.

## Methods

This study consisted of a retrospective review of the video database maintained at the Parkinson’s Disease Center and Movement Disorders Center (PDCMDC) at the Baylor College of Medicine from January 2010 to August 2020. The database includes videos taken of movement disorders patients in our clinic recording studio by clinical professionals (e.g. attendings, fellows, research coordinators) and videos recorded by patients (“selfies”), their friends, family members or care givers in a home, hospital, vehicle, or some other environmental settings that were then provided to the PDCMDC. The term “home” video is used colloquially and applies to all or any of these situations. Videos were catalogued in a “video log” that documented the patient’s age, brief clinical history, phenomenology of movements seen on the video, the patient’s diagnosis, and any ongoing plan of care at the time the video was reviewed by clinicians. Phenomenology, diagnosis, and plan of care were determined by consensus of movement disorders clinicians at regularly scheduled video conferences where patient videos, patient history of present illness, and findings based on physical examination were discussed. We screened the video log for patients who met the following criteria: presented for a clinic visit in which they underwent a formal clinical evaluation comprising of a history of present illness and neurological examination, had a formal video recording in our clinic studio according to our standardized protocol, had a home video of their movement disorder, and consented to allow for both clinic and home videos to be used for research purposes.

In patients who met the criteria for inclusion, a retrospective review of the electronic medical record and video log was performed. Information was gathered on patient age at the time the video was obtained, the patient’s diagnosis, phenomenology of movement disorders noted on clinical examination by the patient’s attending, the phenomenology noted on the video obtained in the clinic determined by PDCMDC attending consensus at video conferences, and the phenomenology of patient home videos determined by PDCMDC attending consensus at video conferences.

Following the retrospective review, home videos were classified as supportive of the diagnosis or not supportive of the initial diagnosis. Home videos were determined to be supportive of a patient’s initial diagnosis if they demonstrated a phenomenology consistent with their diagnosis and/or a response to treatment that would be expected based on their clinical diagnosis. Home videos that did not demonstrate a phenomenological finding, showed a phenomenological finding and/or treatment response inconsistent with the proposed diagnosis, or were of too poor quality to interpret based on the video log consensus were determined to be of no additional value. Home videos were also classified as having provided additional clinical value or being of no additional clinical value. Home videos that provided additional clinical value demonstrated a phenomenology and/or a treatment response that was not or could not be appreciated during their clinical encounter. Home videos of no additional clinical value demonstrated phenomenology or a response to treatment that was also appreciated during the patient’s clinical encounter or met the criteria for videos not supportive of the initial diagnosis as stated above.

Patient home videos were then retrieved from the PDCMDC video database to be reviewed by “blinded” raters. Patient identifying information was removed via Pinnacle video editing software. In cases where patients presented with two or more home videos on a visit, all the home videos were compiled into one video file for review and rating. Blinded raters consisted of 3 movement disorder faculty/attendings and 3 movement disorder fellows at the PDCMDC. Attendings and fellows were asked to identify any attributes that may have interfered with video interpretation. These included any issues with framing of the subject or symptom of interest, sound interference from ambient noise, persons or objects obstructing the camera, poor image quality (i.e. low resolution), inadequate length of video recording, issues with lighting (i.e. too dark, too bright), video stability (i.e. camera shake), poor sound quality (i.e. low volume, sound distortion), and poor camera focus (i.e. blurriness). Based on these attributed they were asked to assign an overall grade to video quality of “poor”, “fair”, “good”, or “excellent”.

Attendings and fellows were then asked to list any applicable phenomenology observed in the video and list the top three most likely clinical diagnoses based on the videos alone. The raters were also asked to assign a confidence level to their interpretation on a scale of 0–100%. No fellow or attending rated a video they had previously viewed during weekly video rounds. Due to this requirement, the number of videos evaluated by each rater in the attending group varied.

Rater responses were compared with the phenomenology and diagnosis as listed in the patient’s chart, and the video log to assess whether raters could accurately assess phenomenology and propose a diagnosis on video alone. As some videos demonstrated multiple phenomenologies, a correct phenomenological diagnosis was defined as one or more phenomenology survey responses matching one or more phenomenologies listed in the corresponding video log entry for the applicable home video even if other phenomenological responses from the same survey were not listed in the video log. An incorrect phenomenological diagnosis was defined as not having identified any phenomenology listed in the video log.

### Statistical analysis

Descriptive statistics were used for evaluation of demographic information and evaluation of utility of patient home videos. Binary logistic regression was used to determine if obtaining a correct phenomenological diagnoses or not could be predicted based on video quality. Chi-square and Fisher’s exact test (when appropriate) were used to assess for associations between video attributes and phenomenology accuracy. Fleiss’ kappa was calculated to measure interrater reliability among the physicians who interpreted all videos. Statistical analysis was performed in SAS studio.

## Results

### Video demographics and clinical utility

A review of the video log identified 20 patient encounters between January 2010 to August 2020 where a home video accompanied a video evaluation performed in the clinic recording studio. The demographic information, identified phenomenologies, and patient diagnoses are listed in ***Table 1***.

**Table 1:** Patient demographics and video characteristics.


**Mean Age ± SD**	35 ± 17.68

**Sex**	11 Male, 9 Female

**Mean video duration ± SD**	91 sec ± 67.98

Diagnoses (n)*	FMD [6], cerebral palsy [3], PD [2], static encephalopathy [2], Tourette syndrome [2], Angelman’s syndrome [1], episodic ataxia [1], eyelid myokymia [1], PSP [1], tardive dyskinesia [1], undiagnosed familial dystonia and chorea [1]

Organic phenomenology (n)**	stereotypy [5], dysarthria [2], parkinsonian gait [2], rest tremor [2], tics [2], ataxia [1], blepharospasm [1], bradykinesia [1], camptocormia [1], chorea [1], generalized dystonia [1], myokymia [1], myoclonus [1], oro-mandibular dystonia [1], palilalia [1]

Phenomenology in functional patients (n)**	tremor [2], truncal titubation [2], astasia abasia [1], camptocormia [1], hemifacial spasm [1]


* 1 patient carried dual diagnoses cerebral palsy and episodic ataxia. ** Multiple phenomenologies were seen per patient. **Abbreviations**: FMD = functional movement disorder, PD = Parkinson’s disease, PSP = progressive supranuclear palsy, SD = standard deviation.

Based on review of the medical records and the video log, 19 (95%) of the home videos were found to be supportive of the initial diagnosis the patients received during their clinic encounter. The one home video not supportive of the initial diagnosis could not be adequately interpreted due to video poor video quality. We also determined that 10 (50%) videos added an additional value beyond the clinic encounter regarding the patient’s diagnosis or treatment. Of these, 9 (45%) videos showed a phenomenological finding that was not noted during clinic examination, helping to further support the diagnosis. One video (5%) helped influence management decisions by demonstrating that a patient’s dystonia was not responsive to levodopa. Notable examples of these include a home video demonstrating paroxysmal gait changes in a functional patient (***Video 1***), and characterization of a severe tic in a Tourette patient that did not occur during the patient’s clinical encounter (***Video 2***).

**Video 1:** **Functional patient with paroxysmal gait changes**. This video shows a 34-year-old woman who presented for evaluation of episodic weakness of the arms and legs who was diagnosed with functional neurological disorder. The clinic video shows give-way weakness and a normal gait. The home video shows that patient during an episode where she has camptocormia and a shuffling gait.

**Video 2:** **Tourette patient with tics not seen in clinic**. This video shows a 9 year-old-girl who presented for management of Tourette syndrome and troublesome shoulder rolling tics. No tics are seen on clinic video even when the fellow videographer vacates the studio. The home and car video shows the patient in severe discomfort and pain associated with dystonic, shoulder rolling tics and other tics.

### Diagnostic accuracy and video characteristics

A total of 93 responses were received by movement disorders attendings (33 evaluations) and fellows (60 evaluations). Raters were able to accurately identify at least one phenomenological finding based solely on patient home video 62.4% of the time. The average reported confidence by raters in phenomenology was 70.4% (standard deviation (SD) 30.3). Based on home video alone, raters were able to accurately identify a correct clinical diagnosis as their top differential diagnosis 34.4% of the time. The correct clinical diagnosis was listed in the top three differential diagnoses 46.2% of the time. Diagnostic confidence among raters was 62.9% (SD 33.1).

There was no difference in frequency of correct phenomenological diagnosis between attending physicians (60.6%) and fellows (63.3%) (x^2^ = 0.07, p = 0.80). Only one attending physician was able to able evaluate all 20 videos, the remaining two attending physicians were limited to 3 evaluations and 10 evaluations as they had previously viewed the other home videos as part of their clinical duties. There was fair inter-rater reliability between raters (one attending physician, and three fellow physicians) who evaluated all 20 videos (κ = 0.35, SD ± 0.18).

### Characteristics of patient videos

Movement disorders attendings and fellows were asked to identify any attributes and assign videos an overall grade to video quality of “poor”, “fair”, “good”, or “excellent”. The odds of identifying the correct phenomenology were significantly lower in the “poor” quality group compared to “excellent” quality videos, with an odds ratio (OR) of 0.07 (p < 0.05, 95% confidence interval (CI): [0.01–0.72]) (***Table 2***). There was no other significant difference noted between overall video quality and accuracy of phenomenology.

**Table 2:** Patient home video quality and frequency of interfering factors in each quality group as determined by physician ratings.


OVERALL VIDEO QUALITY	PHENOMENOLOGY CORRECTLY IDENTIFIED/n	ODDS RATIO (CI)	p	ATTRIBUTES INTERFERING WITH VIDEO INTERPRETATION								

				FRAMING ISSUES	NOISE INTERFERENCE	OBSTRUCTED VIEW	IMAGE QUALITY	SHORT LENGTH	LIGHTING ISSUES	VIDEO STABILITY	SOUND QUALITY	VIDEO FOCUS

Poor	4/20	0.07 [0.01–0.72]	<0.05	45%	35%	20%	60%	20%	65%	50%	15%	55%

Fair	17/30	0.26 [0.03–2.52]	0.25	60%	3%	3%	13%	20%	27%	30%	7%	20%

Good	31/37	1.03 [0.10–10.50]	0.97	24%	3%	11%	3%	19%	24%	11%	–	19%

Excellent	5/6	–	–	–	33%	–	–	–	–	83%	–	–


The table shows how often raters identified the phenomenology correctly in patient home videos in four film quality categories as determined by the rater. The odds ratios of the “poor”, “fair”, and “good” group are compared to the “excellent” group. The attributes that were determined to interfere with interpretation of the home videos are listed by the frequency in which they appear in each quality group. **Abbreviations**: CI = confidence interval, “–” denotes an absent value.

The video attributes that were identified to interfere with interpretation were assessed independently to determine whether they influenced the accuracy of identifying the correct phenomenology on home video (***Table 3***). Image quality was the only attribute significantly associated with a difference in accuracy in phenomenology (35%), (x^2^ = 6.50, p = 0.01). Other notable differences in accuracy were noted in videos with noise interference and poor sound quality, however these differences did not reach statistical significance.

**Table 3:** Attributes interfering with video interpretation.


VIDEO ATTRIBUTE	n	PHENOMENOLOGY ACCURACY	ACCURACY OF VIDEOS WITHOUT INTERFERING ATTRIBUTE	p

Vertical orientation	56	68%	51%	0.13

Framing issue	37	62%	63%	0.97

Noise interference	9	33%	66%	0.08

Obstructed view	9	56%	63%	0.72

Image quality	17	**35%**	**68%**	**0.01**

Short length	19	58%	64%	0.65

Lighting issue	30	60%	63%	0.75

Video stability	23	48%	67%	0.10

Sound Quality	5	20%	65%	0.06

Video focus	24	54%	65%	0.33

None	20	65%	61%	0.78


Video attributes that interfered video interpretation are listed along with how often they were identified, and the accuracy of the phenomenology identified in the video when those attributes were present. This was compared to the phenomenology of videos where that attribute was missing to determine whether there was a significant difference. Phenomenology was significantly lower in videos with poor image quality. No other significant difference was noted.

## Discussion

Film and video recordings of movement disorders patients have a long standing history, and have been used as an adjunct to the physical examination and as a teaching tool as early as the 1920s [8]. Our findings support the many studies that have found utility in video evaluations in the field of movement disorders. Telemedicine evaluations of common scales used in movement disorders have been found to be non-inferior to in-person evaluations [6]. One recent prospective study was able to validate a modified Scale for the Assessment and Rating of Ataxia (SARA) self-recorded by ataxia patients in their own homes [9]. They found that the concordance rate between SARA scores performed in person highly correlated with SARA scores obtained on home videos and evaluated by a trained examiner (r = 0.985, p < 0.0001). These studies, however, utilized highly structured formats, with patients and raters receiving training and detailed instructions or technical advice, such as use of advanced video cameras, tripods, and other professional equipment and systems to produce technically satisfactory recordings. There are limited studies in the field of movement disorders that have evaluated patient home videos specifically. One study describes the use of home videos as an adjunct tool for diagnosis in patients with oro-mandibular dystonia using a multilingual website but it did not evaluate the utility of these videos in clinical diagnosis or treatment [10].

Our study is the first to quantify the value of home videos in determining the phenomenology and diagnosis. Though our sample size was limited, we were able to demonstrate that the majority of home videos taken by patients are of diagnostic value. Indeed, in 50% of cases the home videos provided additional clinical information that would have otherwise not been appreciated during a clinical encounter and in 45% the videos showed a phenomenological finding that was not noted during clinic examination. While our physicians were able to accurately identify a correct phenomenology 62.4% of the time, they were able to determine a correct diagnosis 34.4% of the time based on video alone. We also demonstrated that there is a relationship between the quality of the video and the odds that the physician would be able to interpret it correctly. As such, even though home videos are usually done with cell phones, the recordings can still be highly valuable, especially if patients and their family members or friends are provided instructions on how to best optimize video quality (***Table 4***).

**Table 4:** Recommendations for optimal home video recordings.


If filming with a phone, always use the main camera (rather than the selfie camera) to ensure high quality video Make sure the room or area is well lit Do not cover the camera lens with your fingers During recording the device should be positioned horizontally (not vertically) Make sure the microphone/sound volume is set at maximum Whenever possible, it is best for a second party to film rather than the patient film themselves. Make sure to keep enough space between you in the subject so their whole body (or area of interest) is in the frame Make sure the subject is in focus before filming Focus on capturing the abnormal movement of interest Keep the camera steady and, if possible, use a tripod Do your best to avoid objects (or animals) obstructing the view Minimize ambient noise When possible provide multiple recordings of the same symptom Obtain information how to upload the video into electronic medical records or to the health provider


The inter-rater reliability among our raters who evaluated all videos was fair (κ = 0.35 ± 0.18) and there was no difference in frequency of correct phenomenological diagnosis between attendings and fellows. While retrospective in nature, and without a standardized protocol for filming we still found that our results are comparable to other studies that evaluated the inter-rater reliability of movement disorders experts in recognizing phenomenology by videos obtained by clinicians. In a study by Van der Salm et al., videos of 60 patients with either myoclonus, tics, or psychogenic jerks were evaluated by 39 experienced movement disorder specialists. The inter-rater agreement among experts based on video interpretation alone was fair for myoclonus (κ = 0.29 ± 0.13), psychogenic jerks (κ = 0.22 ± 0.16), and tics (κ = 0.32 ± 0.18) [11]. Another study by Morgante et al. had eight movement disorders experts assess videos of 14 patients with paroxysmal movement disorders and asked them to make a dichotomous judgement on whether the video represented a functional or organic movement disorder. The inter-rater agreement among the experts in this study was also fair (κ = 0.40 ± 0.06) [12]. The discordant accuracies of phenomenologic and etiologic diagnoses, coupled with the fair interrater reliability seen in our and other studies, highlight the limitations of video interpretation in movement disorders, and emphasize the importance of all aspects of the clinical encounter (history of present illness, examination, etc.) in the comprehensive evaluation of a patient in order to arrive at a correct diagnosis.

Where home videos have been found to be particularly useful, with high inter-rater agreement, is in the recognition of functional movement disorders. In a study that evaluated whether or not functional neurological disorders could be identified by videos in the news media, 10 movement disorder specialists were asked to identify 7 cases that were presumed to be functional movement disorders and 1 control case with an organic diagnosis [13]. All 10 experts were able to agree on a functional movement disorder diagnosis in 6 out of 7 presented cases with 9 out of 10 experts agreeing on the 7^th^ case. The overall inter-rater reliability including the control case was very good (κ = 0.89, 95% CI: 0.79–1.00). Another study asked 7 movement disorder neurologists to evaluate 29 videos of movement disorders on YouTube to judge whether not the video represented a functional or organic disorder [14]. They identified 66% of the videos to be functional with an excellent inter-rater agreement (κ = 0.89). Movement disorder clinicians have also used home videos posted on social media as a diagnostic tool in identifying functional tics in other patients [15]. A recent case series described 6 teenage girls (mean age 14.2) who developed abrupt onset tic movements that were ultimately determined to be functional in nature. All 6 reported watching videos of tics in same social media personality on the platform TikTok prior to onset of their symptoms. Aside from other typical features of functional movement disorders, one additional clue as to the diagnosis was the presence of specific movements and sounds that were demonstrated by the social media personality and replicated by the patients in the cohort. Since that report we have encountered over 70 teenage girls with these TikTok tics over the past year, who have viewed and posted their home videos on various social media platforms. This phenomenon is similar to “conversion disorder or mass psychogenic illness” reported in the past [16]. Due to our limited sample size we were not able to adequately assess the utility of home videos in this patient population however this may be the area where their use could be most beneficial.

We recognize several limitations in our study. Due to the need to preserve the blind, raters were excluded from rating if they previously reviewed the videos during weekly video rounds. Therefore, not every video was evaluated an equal number of times by raters. We also recognize that the patient disease demographics are not representative of a typical population in a movement disorders practice and, therefore, the findings may not be generalizable. This may be due to selection bias for rarer conditions or a higher frequency of atypical or functional movement disorder requiring second opinion based on video review. Conversely, these demographics may better represent those complex patients in whom supplemental home video recording can be more helpful. Other limitations include small sample size and the retrospective nature of the study.

## Conclusion

Home video recordings allow for a unique and additional perspective and remain a vital tool in evaluation of patients with organic and functional movement disorders. Several barriers to more routine usage may be technological constraints on the patient or the providers office and poor integration of video sharing and the electronic medical record. As it is well recognized that telemedicine and remote medicine will continue to become more common place, we think it is important to encourage high quality home videos as a supplemental tool in diagnosis and management of patients with movement disorders.
